# Comparing SARS-CoV-2 variants among children and adolescents in Germany: relative risk of COVID-19-related hospitalization, ICU admission and mortality

**DOI:** 10.1007/s15010-023-01996-y

**Published:** 2023-02-14

**Authors:** Marietta Jank, Anna-Lisa Oechsle, Jakob Armann, Uta Behrends, Reinhard Berner, Cho-Ming Chao, Natalie Diffloth, Maren Doenhardt, Gesine Hansen, Markus Hufnagel, Fabian Lander, Johannes G. Liese, Ania C. Muntau, Tim Niehues, Ulrich von Both, Eva Verjans, Katharina Weil, Rüdiger von Kries, Horst Schroten

**Affiliations:** 1 Department of Pediatrics, University Medical Center Mannheim, Heidelberg University, Theodor-Kutzer-Ufer 1-3, 68167 Mannheim, Germany; 2 Department of Pediatric Surgery, University Medical Center Mannheim, Heidelberg University, Theodor-Kutzer-Ufer 1-3, 68167 Mannheim, Germany; 3 Division of Pediatric Epidemiology, Institute of Social Pediatrics and Adolescent Medicine, Ludwig-Maximilians-University Munich, 80336 Munich, Germany; 4 Department of Pediatrics, University Hospital and Medical Faculty Carl Gustav Carus, TU Dresden, Dresden, Germany; 5 Department of Pediatrics, Faculty of Medicine, Technical University Munich, 80804 Munich, Germany; 6 Department of Pediatrics, Helios University Medical Center, Witten/Herdecke University, Heusnerstr. 40, 42283 Wuppertal, Germany; 7 Cardio-Pulmonary Institute (CPI), Universities of Giessen and Marburg Lung Center (UGMLC), German Center for Lung Research (DZL), Justus-Liebig University Giessen, Giessen, Germany; 8 Centre for Pediatrics and Adolescent Medicine, Hannover Medical School, Excellence Cluster RESIST, Deutsche Forschungsgemeinschaft (DFG), EXS 2155, 30625 Hannover, Germany; 9 Department of Pediatrics and Adolescent Medicine, Medical Faculty, University Medical Center, University of Freiburg, Freiburg, Germany; 10 Division of Paediatric Infectious Diseases, Department of Pediatrics, University Hospital of Wuerzburg, 97080 Würzburg, Germany; 11 Medical Center Hamburg-Eppendorf, University Children’s Hospital, Martinistrasse 52, 20246 Hamburg, Germany; 12 Department of Pediatrics, Helios Klinikum Krefeld, 47805 Krefeld, Germany; 13 Dr von Hauner Children’s Hospital, University Hospital, Ludwig-Maximilians-University, 80337 Munich, Germany; 14 Department of Pediatrics, Medical Faculty, University Hospital RWTH Aachen, 52074 Aachen, Germany; 15 Department of General Pediatrics, Neonatology, and Pediatric Cardiology, Medical Faculty, University Hospital, Heinrich-Heine-University Düsseldorf, 40225 Düsseldorf, Germany

**Keywords:** COVID-19, Pandemic, Children, Adolescents, SARS-CoV-2, Variants of concern, Seroprevalence, Burden of disease

## Abstract

**Purpose:**

SARS-CoV-2 infections cause COVID-19 and have a wide spectrum of morbidity. Severe disease courses among children are rare. To date, data on the variability of morbidity in relation to variant of concern (VOC) in children has been sparse and inconclusive. We compare the clinical severity of SARS-CoV-2 infection among children and adolescents in Germany during the Wildtype and Alpha combined, Delta and Omicron phases of the COVID-19 pandemic.

**Methods:**

Comparing risk of COVID-19-related hospitalization, intensive care unit (ICU) admission and death due to COVID-19 in children and adolescents, we used: (1) a multi-center seroprevalence study (SARS-CoV-2-KIDS study); (2) a nationwide registry of pediatric patients hospitalized with SARS-CoV-2 infections; and (3) compulsory national reporting for RT-PCR-confirmed SARS-CoV-2 infections in Germany.

**Results:**

During the Delta predominant phase, risk of COVID-19-related hospitalization among all SARS-CoV-2 seropositive children was 3.35, ICU admission 1.19 and fatality 0.09 per 10,000; hence about halved for hospitalization and ICU admission and unchanged for deaths as compared to the Wildtype- and Alpha-dominant period. The relative risk for COVID-19-related hospitalization and ICU admission compared to the alpha period decreased during Delta [0.60 (95% CI 0.54; 0.67) and 0.51 (95% CI 0.42; 0.61)] and Omicron [0.27 (95% CI 0.24; 0.30) and 0.06 (95% CI 0.05; 0.08)] period except for the < 5-year-olds. The rate of case fatalities decreased slightly during Delta, and substantially during Omicron phase.

**Conclusion:**

Morbidity caused by SARS-CoV-2 infections among children and adolescents in Germany decreased over the course of the COVID-19 pandemic, as different VOCs) emerged.

**Supplementary Information:**

The online version contains supplementary material available at 10.1007/s15010-023-01996-y.

## Introduction

Disease burden related to SARS-CoV-2 infection among children and adolescents has proven to be substantially lower than among adults [1]. Evidence for the mainly asymptomatic and benign course of acute pediatric SARS-CoV-2 infection first emerged during an outbreak investigation of the Wildtype variant in Iceland during April 2020 [2]. Since then, this tendency has been confirmed by a number of analyses [3]. At the same time, however, Pediatric Inflammatory Multisystem Syndrome associated with COVID-19 (PIMS-TS, also known as MIS-C)—a complex, auto-inflammatory, SARS-CoV-2-associated condition that requires intensive care unit (ICU) admission in nearly 50% of cases—has emerged as the main COVID-19 pandemic-related morbidity among children and adolescents [4].

Recent data show that the risk for PIMS-TS steadily decreased in parallel with the successive emergence of Delta and Omicron variants of the SARS-CoV-2 virus [5]. One of the earliest variant of concern (VOC) was Alpha (B.1.1.7), which was first detected in the UK and a short time later (December 2020) in Germany. More transmissible than the previous Wildtype variant, Alpha accounted for over 50% of sequenced isolates by the end of June 2021 [6]. Following Alpha, the highly contagious Delta variant, which first emerged in India in December 2020, rapidly became the predominant variant circulating in Germany from summer through December 2021, when Omicron began emerging [7].

Early studies in adults showed the Delta variant to be linked to an increased risk of hospitalization as compared to earlier VOCs [8–10]. To date, evidence regarding disease severity of COVID-19 among children and adolescents infected with different VOCs has been both limited and controversial [9–14]. Regarding Omicron variants, current data suggest that need for hospitalization varies by age group [15].

In the present analysis, the COVID-19-related burden of disease among children and adolescents in Germany during the Delta and Omicron period is compared with the Wildtype/Alpha period as a reference.

## Materials and methods

For our analysis, we used data from the following three sources: (1) the SARS-CoV-2 KIDS study; (2) the German Society for Pediatric Infectious Diseases (DGPI) registry; and (3) the statutory notification system of the Robert Koch Institute (RKI), Germany’s national public health institute.

The SARS-CoV-2 KIDS study is a hospital-based, multicenter, longitudinal study collecting data from children aged ≤ 17 years in Germany. Methods have been previously published [16]. In brief, the method was unchanged, except for explicit exclusion of children with prior vaccination against SARS-CoV-2. The recruitment period was extended from June 2021 to October 2021 in 12 participating pediatric hospitals. This yielded data from an additional 1,885 participants.

Seroprevalence estimates included in the SARS-CoV-2 KIDS study publication were extrapolated to the total population of children in Germany using data provided by the German Federal Statistical Office [17].

In March 2020, the German Society for Pediatric Infectious Diseases (DGPI) initiated a national, hospital-based, prospective registry to report symptomatic and asymptomatic cases of children and adolescent hospitalized with SARS-CoV-2 infection. Data collected included demographic information, symptoms and clinical signs, treatment (including need for intensive care), disease course during hospitalization, and outcome at time of hospital discharge. Only cases of hospitalized children receiving COVID-19-related treatments were counted in the analysis.

The third source of data analyzed came from the nationwide, state-based reporting system for laboratory-confirmed SARS-CoV-2 infections in Germany. Laboratories and physicians are legally obliged to report detection of SARS-CoV-2 nucleic acid by reverse transcription polymerase chain reaction (RT-PCR) or culture isolation to public health authorities. This information is then relayed to the Robert Koch Institute (RKI—Germany’s national public health institute). Data on hospitalizations and deaths in the German pediatric population overall, as well as in the age groups defined by our study, was kindly provided by the RKI.

### Outcome measurements/endpoints

To establish outcome measurements, we considered the following: (1) hospital admission for COVID-19-related treatment, as defined by the physician who reported the case to the DGPI registry; (2) admission to an ICU due to COVID-19-related symptoms, as reported to the DGPI registry; and (3) death associated with COVID-19, as reported to the national, state-based reporting system for RT-PCR-confirmed SARS-CoV-2 infections in Germany.

We defined severe COVID-19 by (1) the need of hospitalization for COVID-19-related treatment, (2) admission to an ICU or (3) death due to COVID-19.

### Definition of VOC waves

In Germany, distribution of predominant SARS-CoV-2 variants is monitored by the RKI, which makes the data publicly available. On the basis of this data, we defined three pandemic periods according to calendar week (CW) as follows: (1) the Wildtype- and Alpha-dominant VOC wave: CW9 2020-CW24 2021, i.e., March 2020 to June 2021; (2) the Delta-dominant VOC wave: CW25-CW52, i.e., July to December 2021; and (3) the Omicron-dominant VOC wave: CW1-CW16, i.e., January to April 2022. The dominant variant was defined as that which accounted for > 50% of SARS-CoV-2 infections during the respective CW.

### Statistical analysis

To calculate risk for the severity outcomes described above, we estimated the number of SARS-CoV-2-infected children using population demographics provided by the German Federal Statistical Office and the SARS-CoV-2 KIDS Study data.

Taking into account sampling imprecision, we used the upper and lower limits of the reported 95% confidence interval (CI) as a denominator for the risk estimate limits for the outcome measurements.

In alignment with German COVID-19 vaccination recommendations, risks calculated were stratified by age group. The age groupings were: < 5 years old, 5–11 years old, and 12–17 years old. At first (in June 2021), COVID-19 vaccination for children in Germany were only recommended for 12 to 17-year-olds with pre-existing conditions [18]. In August 2021, the recommendation was extended to include all 12–17-year-olds [18]. In January 2022, a vaccine for children aged 5–11 years old became available. Initially, this was recommended only for children with pre-existing conditions [19]. In May 2022, the recommendation was extended to include all children > 5 years [19].

Since the DGPI registry was based on voluntary reporting by pediatricians, we are unable to assume complete reporting. To correct for underreporting in our statistical analyses, we multiplied the number of COVID-19-related treatments and ICU admissions by the rate of the respective underreporting in hospitalization numbers. From July 2021 to October 2021, 388 children were reported in the DGPI registry and 2169 to the state-based reporting system. From July to October 2021, the underreporting rate was 3.99 for < 5-year-olds, 9.07 for 5 to 11-year-olds, and 7.64 for 12 to 17-year-olds, respectively. Further rates for the respective periods can be found in Table 1 of the Appendix. Concerning death, we used data provided by the RKI, and assumed this data to be complete. For this reason, we did not correct for underreporting regarding mortality.

Because the seroprevalence study ended on October 31, 2021, no absolute risk for severe COVID-19 manifestations could be estimated for the period November 2021–April 2022.

To extend our analysis to cover the Omicron period, we used the number of RT-PCR reported SARS-CoV-2 infections as a denominator. By dividing the number of patients hospitalized for COVID-19 and COVID-19-related ICU admissions by the number of RT-PCR-confirmed SARS-CoV-2 infections, risks per 10,000 SARS-CoV-2 infections were calculated with a 95% CI (Table 2).

Statistical analysis was performed using SAS Version 9.4 (SAS Institute, Cary, NC, USA). Significance level was defined as 5%.

### Ethics committee approvals

Data was collected in accordance with the principles of the declaration of Helsinki and approval of local ethics committee was obtained.^1^

## Results

As of December 31, 2021, the population children and adolescents of  ≤ 17 years in Germany was approximately 13.9 million. Based on the estimates derived from the SARS-CoV-2 KIDS study, the seroprevalence in the pediatric population ≤ 17 years in July 2021 was 14.04% (95% CI 10.66; 17.42). This increased to 21.67% (95% CI 16.01; 27.34) in October 2021, which suggests that at least 1,057,767 (95% CI 741,684; 1,375,235) children ≤ 17 years of age were infected during this period.

**Fig. 1:**
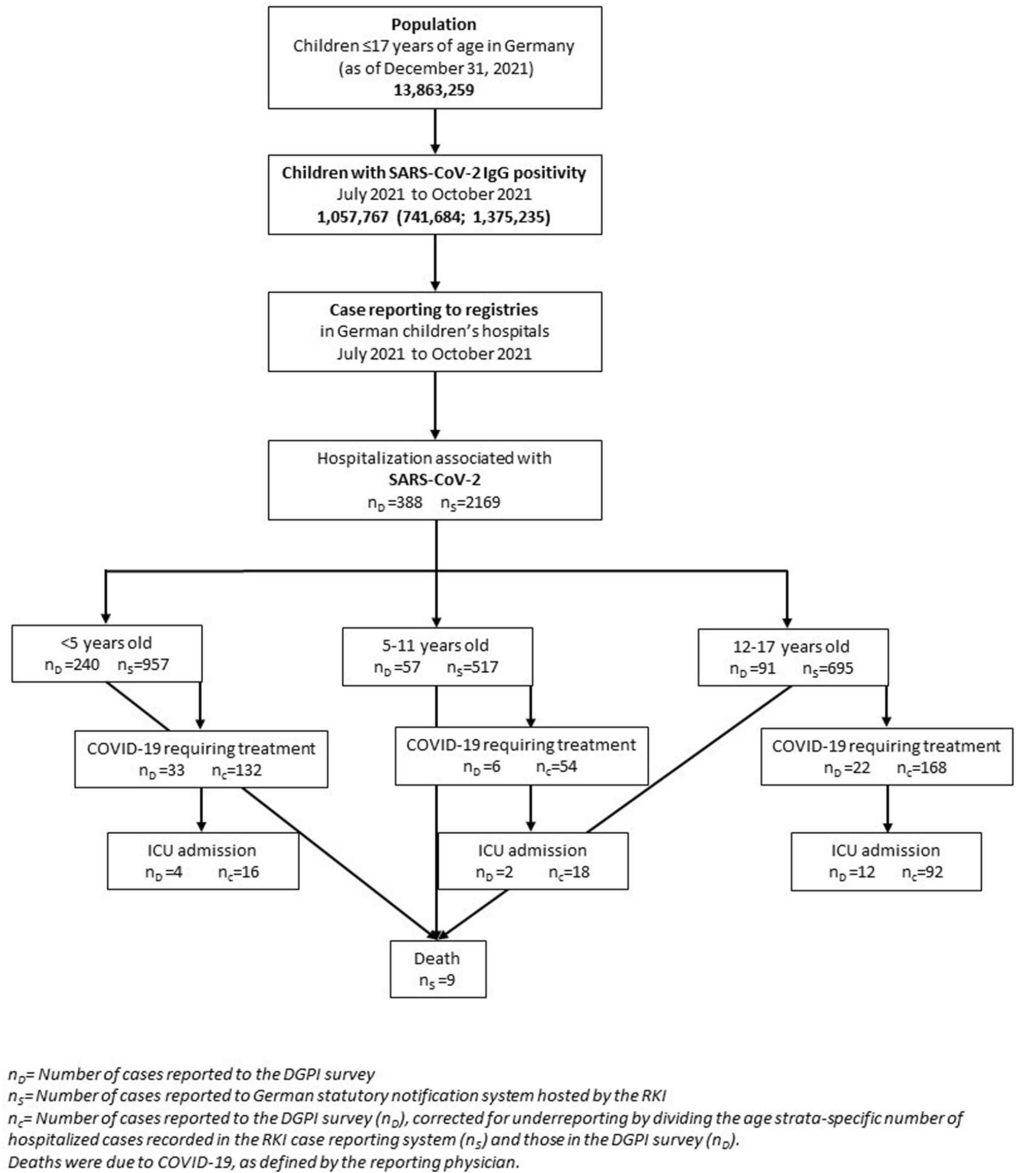
Flowchart displaying reported pediatric COVID-19 cases with COVID-19-related hospitalization, ICU admission and death from July to October 2021

The flowchart in Fig. 1 shows the number of children hospitalized with SARS-CoV-2, as reported to the DGPI registry (n_D_). However, according to the state-based reporting system (n_S_), the number of reported hospitalizations was substantially higher, which suggests an underreporting in n_D_. Therefore, the number of SARS-CoV-2 infections requiring COVID-19-related hospitalization or ICU admission in the DGPI registry are in need of correction for underreporting (n_c_): The n_c_ for COVID-19-related hospitalization were 132 in < 5-year-olds, 54 in 5–11-year-olds and 168 in the 12–17-year-olds. Using the same multiplier for underreporting, in total 126 SARS-CoV-2-infected children were admitted to ICUs for COVID-19-related treatment (Fig. 1).

### Comparing the risk for hospitalization, ICU admission and death associated with SARS-CoV-2 infection in children and adolescents, as defined by increment in seropositivity during Wildtype/Alpha and Delta predominant periods

From July to October 2021, a period defined as belonging to the Delta VOC period, overall risk of COVID-19-related hospitalization was 3.35 (95% CI 2.57; 4.77) per 10,000 SARS-CoV-2-infected children and adolescents in Germany. With respect to age group-specific results, the 95% confidence intervals for hospitalization risk overlapped with 4.35 (95% CI 3.35; 6.21) among children < 5 years old, 3.46 (95% CI 2.90; 4.30) among 5–11-year-olds, and 4.88 (95% CI 3.75; 6.96) among 12–17-year-olds.

In comparison with the period, in which Wildtype and Alpha VOC were most prevalent in Germany [5], our new data covering the period July to October 2021, when Delta predominated, shows the risk of COVID-19-related hospitalization to be substantially reduced. Overall, the risk of COVID-19-related ICU admission was 1.19 (95% CI 0.92; 1.70) per 10,000 SARS-CoV-2 infected children; with some variation in the different age groups. These rates are substantially lower than what previously published data for the Wildtype and Alpha period showed [5].

The case fatality rate during the Delta predominant period (July to October 2021) was unchanged compared to the Wildtype and Alpha VOC phase [0.09 (95% CI 0.08; 0.12) vs. 0.09 (95% CI 0.07; 0.12)] (Table 1). In both periods, almost all children who died of COVID-19 had risk factors [20].

**Table 1:** Risk for hospitalization, ICU admission and death associated with SARS-CoV-2 infection in children and adolescents, as defined by increment in seropositivity during alpha and delta predominant periods: Severe COVID-19

Time period	Population at risk^#^	Increment in SARS-CoV-2 IgG positivity	Hospitalization for COVID-19 related therapy		COVID-19 related ICU admission		Death due to COVID-19	
			n_c_	Risk per 10,000 (95% CI)	n_c_	Risk per 10,000 (95% CI)	n_S_	Risk per 10,000 (95% CI)
All								
May 2020–May 2021 ^+^	13,743,944	1,484,346 (1,195,723; 1,772,969)	1059	7.13 (5.97; 8.86)	328	2.21 (1.85; 2.74)	14	0.09 (0.08; 0.12)
July 2021–Oct 2021	13,863,259	1,057,767 (741,684; 1,375,235)	354	3.35 (2.57; 4.77)	126	1.19 (0.92; 1.70)	9	0.09 (0.07; 0.12)
Children < 5 years old								
May 2020–May 2021 ^+^	3,969,138	428,667 (345,315; 512,019)	391	9.12 (7.64; 11.32)	60	1.40 (1.17; 1.74)	8	0.19 (0.16; 0.23)
July 2021–Oct 2021	3,975,333	303,318 (212,680;394,353)	132	4.35 (3.35; 6.21)	16	0.53 (0.41; 0.75)	*	
Children 5–11 years old								
May 2020–May 2021 ^+^	5,267,742	568,916 (458,294; 679,539)	197	3.46 (2.90; 4.30)	88	1.55 (1.29; 1.92)	4	0.07 (0.06; 0.09)
July 2021–Oct 2021	5,375,407	410,144 (287,584; 533,240)	54	1.32 (1.01; 1.88)	18	0.44 (0.34; 0.63)	*	
Adolescents 12–17 years old								
May 2020–May 2021 ^+^	4,507,064	486,763 (392,115; 581,411)	471	9.68 (8.10; 12.01)	180	3.70 (3.10; 4.59)	2	0.04 (0.03; 0.05)
July 2021–Oct 2021	4,512,519	344,305 (241,420; 447,642)	168	4.88 (3.75; 6.96)	92	2.67 (2.06; 3.81)	*	

n_D_ = number of cases reported to the DGPI registry, n_S_ = number of cases reported to German statutory notification system hosted by the RKI, n_c_ = number of cases reported to the DGPI registry (n_D_), corrected for underreporting by dividing the age strata-specific numbers of hospitalized cases recorded in the RKI case reporting system (n_S_) and those in the DGPI registry (n_D_)

^#^Children ≤ 17 years living in Germany; *age-specific mortality risk was not calculated for patient data protection reasons;  + data duplicated from a previously published paper by the same authors [23]

### Comparing the risk of COVID-19-related hospitalization and ICU admission via analysis of SARS-CoV-2 infections recorded in the compulsory statuary reporting system and confirmed by RT-PCR during periods with different predominant VOCs in Germany

Irrespective of age group, the number of RT-PCR-confirmed infections in the German pediatric population increased during the course of the pandemic (Delta: 236,886 and Omicron: 4,088,268 RT-PCR-confirmed infections; Table 2).

**Table 2:** Comparing the risk of COVID-19-related hospitalization and ICU admission via analysis of SARS-CoV-2 infections recorded in the compulsory, statuary reporting system and confirmed by RT-PCR during periods with different predominant VOCs in Germany

Time period	Number of children with RT-PCR-confirmed infection	Hospitalization for COVID-19-related therapy		COVID-19-related ICU admission		Death due to COVID-19	
		n_c_	Risk per 10,000 (95% CI)	n_c_	Risk per 10,000 (95% CI)	n_c_	Risk per 10,000 (95% CI)
All							
March 21–June 21 (CW9-24)	236,886	457	19.29 (17.52; 21.06)	161	6.80 (5.75; 7.85)	5	0.21 (0.03; 0.404)
July 21–December 21 (CW25-52)	923,497	1069	11.58 (10.88; 12.27)	318	3.44 (3.07; 3.82)	16	0.17 (0.09; 0.26)
January 22–April 22 (CW1-16)	4,088,268	2122	5.19 (4.97; 5.41)	180	0.44 (0.38; 0.50)	20	0.05 (0.03; 0.07)
Children < 5 years old							
March 21–June 21 (CW9-24)	51,519	134	26.01 (21.61, 30.41)	26	5.05 (3.11, 6.99)	*	
July 21–December 21 (CW25-52)	114,653	370	32.27 (28.99, 35.55)	84	7.33 (5.76, 8.89)	*	
January 22–April 22 (CW1-16)	621,273	1168	18.80 (17.72, 19.88)	53	0.85 (0.62, 1.08)	*	
Children 5–11 years old							
March 21–June 21 (CW9-24)	96,242	79	8.21 (6.40; 10.02)	55	5.71 (4.20; 7.22)	*	
July 21–December 21 (CW25-52)	471,053	270	5.73 (5.05; 6.42)	100	2.12 (1.71; 2.54)	*	
January 22–April 22 (CW1-16)	1,957,608	472	2.41 (2.19; 2.63)	60	0.31 (0.23; 0.38)	*	
Adolescents 12–17 years old							
March 21–June 21 (CW9-24)	89,125	245	27.49 (24.05; 30.93)	79	8.86 (6.91; 10.82)	*	
July 21–December 21 (CW25-52)	337,791	429	12.70 (11.50; 13.90)	134	3.97 (3.30; 4.64)	*	
January 22–April 22 (CW1-16)	1,509,387	482	3.19 (2.91; 3.48 + 134)	68	0.45 (0.34; 0.56)	*	

nc = number of cases reported to the DGPI survey (nD), corrected for underreporting by a factor based on the division of the age strata-specific numbers of hospitalized cases recorded in the RKI case report and in the DGPI survey

*Age-specific mortality risk was not calculated for patient data protection reasons

**Fig. 2:**
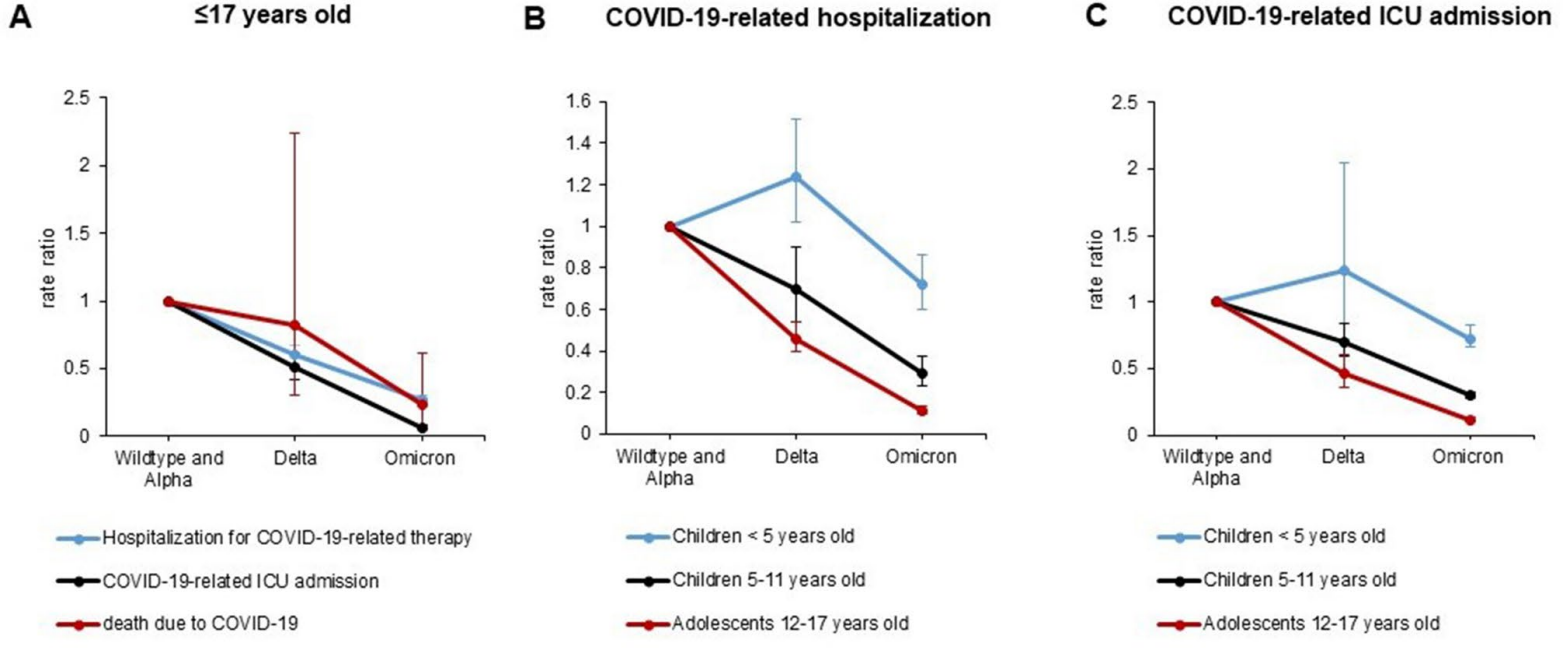
Rate ratios of COVID-19-related hospitalization, ICU admission and death in children ≤ 17 years of age during the periods of the SARS-CoV-2 pandemic, in which Delta and Omicron were predominant with Wildtype/Alpha as reference (**A**). Rate Ratios of hospitalization for COVID-19 related therapy, COVID-19-related ICU admission and death for all age groups combined; the respective rate ratios per age groups are shown in (**B**) for hospitalization and (**C**) for ICU admission

The rate of COVID-19-related hospitalizations decreased among 5–11-year-olds and 12–17-year-olds over the course of the pandemic (March 2021–April 2022), whereas there was a slight increase among < 5-year-olds, with an overlapping 95% CI during the Delta period. During the Omicron period the overall rate of hospitalizations was reduced to 27% compared to the Wildtype and Alpha period (Table 2, Fig. 2).

The rate of COVID-19-related ICU admissions among children and adolescents decreased substantially during the pandemic, with the low point arriving during Omicron phase [0.44 (95% CI 0.38; 0.50) per 10,000] accounting for a reduction to 6.5% compared to the Wildtype and Alpha period. Among the < 5-year-olds during the respective reduction was to 16.8% (Table 2, Fig. 2).

The rate of COVID-19 related deaths decreased slightly with overlapping 95% CI’s during the Delta period; while during Omicron there was clear decrease in death rates to 23.3% compared to the Wildtype and Alpha period with no overlapping 95% CI’s compared to the Delta period (Table 2, Fig. 2).

## Discussion

COVID-19-related morbidity, already substantially lower among children and adolescents than among adults with the Wildtype variant, further decreased during the course of the pandemic. The pattern observed was similar to that for PIMS-TS, with a reduction in ICU admissions going down to approximately 10% of the rate reported during the Wildtype/Alpha period [4].

Similar observations regarding COVID-19 morbidity have been made in relation to the populations of other countries [21, 22]. However, these reports for the most part have not been based upon risk estimates that apply a denominator defined by serologic testing [22]. The advantage of considering serologic testing when analyzing such data is that it allows for identification of almost all SARS-CoV-2 infections, regardless of symptoms [16]. However, when the same testing strategy is applied during the whole observation period, times series based upon RT-PCR reports may be equally valid in depicting trends over time. Calculation of absolute risks will not be possible, though. Screening in kindergartens and schools was conducted during the whole observation period (March 2021–December 2022) [23, 24]. Based upon RT-PCR, the absolute incidence estimates at each time point are higher than they are with a denominator-based seroprevalence increment given the degree of underreporting in the statutory reporting system.

The increase in seropositivity prevalence estimates in our study is consistent with previously published UK data which shows a large increase in seropositivity during the period September to October 2021, following a stable plateau in the summer of 2021 [25]. This may be due to the higher transmissibility of Delta as compared to Alpha [9, 26]. It also coincides with the return to school of 5–17-year-old children, most of them unvaccinated at that time [27, 28].

Data published regarding the severity of COVID-19 infections during the Delta and Omicron periods are inconclusive. Data from the US National COVID Cohort Collaborative show a decrease in severe disease as compared to moderate disease during the Delta phase, while also reporting similar hospitalization rates during the Delta and Omicron periods [12]. During the Delta period in the UK, school-aged children were hospitalized with COVID-19 at rates similar to those during the Alpha period [29]. In contrast to these results, data from a nationwide COVID-19 registry in Japan described an increase in the number of pediatric patients admitted to intensive care units during the Delta period vs. the pre-Delta period [13]. In this Japanese study, however, the proportion of pediatric patients with underlying disease was higher during the Delta period, when 50% of those admitted to ICU had comorbidities. This may explain the high ICU admission rate detected by this study for the Delta period. Nevertheless, similar findings were described by a Scottish study which showed a doubled risk of COVID-19 hospitalization during Delta period. This study also highlighted children with comorbidities as a risk group [30].

With regard to severity of disease, during the Delta period, our data showed an overall decrease in morbidity among children and adolescents in Germany: COVID-19-related hospitalizations and ICU admissions, irrespective of which denominator was applied for the risk estimates. In the < 5-year-old group, there was a slight discrepancy regarding the risk of COVID-19-related hospitalization and ICU admission, as defined by the increment in seropositivity in comparison to the RT-PCR testing numbers in our study. Here, we noted an increased risk during the Delta period when considering RT-PCR data. One possible explanation for this is the lower number and reliability of routine testing methods (i.e., lolly or spit tests) employed for this age group, as compared to those used for children > 5 years old—a difference that may result in a lower detection of asymptomatic SARS-CoV-2 infections. Older children attending educational institutions were tested according the nationwide recommendations in March 2021 through March 2022. Even though health care in Germany is primarily in the responsibility of the states, during the pandemic there have been nationwide recommendations [31]. However, the local test strategies possibly varied e.g., with some institutions performing pooled PCR tests and others applying a sequential approach (antigen testing followed by confirmation in RT-PCR). This aspect further underlines the importance of seroprevalence surveys when evaluating disease severity for this age group.

With emergence of Omicron in Germany, the number of SARS-COV-2 infections increased rapidly among children and adolescents. However, whereas the absolute numbers of reported COVID-19-related hospitalization increased, ICU admission numbers remained stable. Risk estimates based upon RT-PCR-confirmed SARS-COV-2 infections decreased in comparison to the Alpha and Delta periods. This decrease was most pronounced in connection with ICU admissions—results which are in line with previous findings and which confirm Omicron as being associated with less severe disease than Delta [15, 32]. Although data from a population-based surveillance survey for laboratory-confirmed, COVID-19-associated hospitalizations in the United States (COVID-NET) showed there to have been a higher hospitalization rate among children during the Omicron phase as compared to the Delta phase, a lower proportion of these patients required ICU admission or mechanical ventilation [33]. Consistent with our findings, Whittaker et al. describe a lower risk of hospitalization in connection with acute COVID-19 among < 18-year-olds in Norway during the Delta and Omicron periods, as compared to during the Alpha period [11].

Studies in adults [34–37] found an increased risk of hospital admission and severe COVID-19 during the Delta period compared to preceding variants (Wildtype and Alpha). Omicron was associated with less severe disease but still resulted in substantial morbidity and mortality, especially in risk groups. Vaccinated patients admitted for COVID-19-related treatment experienced significantly lower disease severity regardless of infecting variant.

Increased concern about the new condition, might prompt more hospital admissions in the early phase of the COVID-19 pandemic. Literature, however, suggests that an increased fear of possible infection with SARS-CoV-2, resulted in a reduction of medical consultation and hospital admission for respiratory infections [38–40].

Current recommendation for clinical practice in COVID-19 treatment for children in Germany are mainly based on extensive studies in adults [41]. Furthermore, only a minority of children and adolescents have a severe disease course requiring specific anti-inflammatory (e.g. dexamethasone) or anti-viral medication (e.g. monoclonale antibodies, such as Sotrovimab). Therefore, we think the evolving medical approaches in COVID-19 treatment plays a minor role in the morbidity of SARS-CoV-2 Infection the whole pediatric population.

COVID-19 vaccination in children provides protection against severe Omicron infection, resulting in lower hospitalization rates [42–44]. Vaccination and higher naturally acquired immunity due to circulation of Omicron variant and pediatric COVID-19 vaccination campaigns in Germany may contribute to decreasing risk for hospitalization and ICU admission among children > 5 years olds due to a dramatically decreasing immune-naïve children [15]. However, vaccination for children aged < 5-years-old was not available during the observation period. Therefore, decrease in morbidity in this age group would best reflect the effect of lower pathogenicity of Omicron. Interestingly, in our data a substantial reduction in COVID-19-related hospitalization and ICU admission was observed in this age group.

Recent virological studies show a decreased pathogenicity of the Omicron variant due to its unique features, such as a higher replication competence in the bronchi compared to the lung resulting in a robust upper respiratory tract infection, but less severe lower respiratory tract disease [45]. Furthermore, Omicron shows a lower fusogenic potential and less efficient cleavage of the spike protein in comparison to previous VOCs [46].

### Strength and limitations

In its analysis of COVID-19 disease severity among children and adolescents in Germany, the strength of our study lies in its basis on seroprevalence increments data and RT-PCR-based time series. For the pediatric population, this is of particular concern, as nearly half of all COVID-19 infections in children are asymptomatic [25]. In the context of our previously published data [5, 16], which uses the same data sources and algorithms, our current study is able to describe the risk of hospitalization and ICU admission during the different VOC periods.

Despite these advantages, several potential limitations need to be considered regarding the infection rate estimates which provide the denominator for the risk estimates' potential sources of bias: seroprevalence and reported RT-PCR-confirmed infections. First, of note, seroprevalence is susceptible to sampling bias—a factor that needs to be considered as the number of samples recruited decreased during the extension period of SARS-CoV-2 KIDS study, (i.e., July 2021-October 2021). Furthermore, due to the waning of antibodies over time, we are unable to identify all preceding infections. However, findings suggest that SARS-CoV-2 antibodies in children may persist for over a year after infection [47, 48].

Furthermore, when we started the SARS-CoV-2 KIDS-Study in March 2021 commercially available ELISA kits were restricted to detecting S-Protein and the data published in this manuscript was an extension of the SARS-CoV-2 KIDS-study, hence we only determined S-Protein antibodies. Therefore, we could not validate the children’s reported vaccination status.

Although the RKI data have limitations as to whether the PCR-positive children had been hospitalised because of or with SARS-CoV-2 infection the data could be used to validate completeness of infections in hospitalised children because the case definition for reporting to the DGPI registry was identical. Since detailed clinical information was available for cases reported to the DGPI registry, children with COVID-19 disease could be identified based on whether they received COVID-19-related therapy.

Increasing vaccination rates might result in herd immunity, especially among adolescents > 11 J. Thus, the population at risk for infection might be overestimated based on the census data accounting for underestimation of the risk for COVID-19-related hospitalisation during Omicron. However, while protection from vaccination against severe disease was excellent during Omicron phase, protection against infection was poor. Therefore, we are convinced that the size of this bias should be small.

## Conclusion

Net morbidity related to COVID-19 among children and adolescents in Germany showed a steady decrease with the emergence of each new variant during the pandemic. These findings may provide important guidance when establishing recommendations for vaccinating children during late phases of the pandemic, as we near the time when the virus becomes endemic.

## Supplementary Information

Below is the link to the electronic supplementary material.

Supplementary file1 (DOCX 30 KB)

## Data Availability

Data and analysis code can be requested from the corresponding author upon reasonable request.

## Footnotes

1 The DGPI registry was approved by the ethics committee of the Technical University Dresden (Nr. BO-EK-110032020). The ethics committee of the Medical Faculty of Heidelberg University (Nr. 2020- 536N) provided an initial evaluation of the SARS-CoV-2 KIDS study. Subsequently, the ethics committees of the participating hospitals independently approved the study protocol: Ethics Committee of the Medical Faculty of the Ludwig-Maximilians-University Munich Nr. 20–348, Technical University Dresden Nr. BOEK142042020, Medical Faculty of the HHU Düsseldorf Nr. 2020–936, Hamburg Medical Association Nr. MC-142/20, Nordrhein Medical Association Nr. 2,020,099, Albert-Ludwigs-University Freiburg Nr. 243/20, Medical Faculty of the RWTH Aachen Nr. 081/20, Medical Faculty of the Justus-Liebig University Giessen Nr. 61/20, MMH Hannover Nr. 9041_BO_K_2020, Julius-Maximilians University Würzburg Nr. 92/20_z, Technical University Munich Nr. 264/20S.
