# Supplementary material for: Comparing SARS-CoV-2 variants among children and adolescents in Germany: relative risk of COVID-19-related hospitalization, ICU admission and mortality
Source: Infection. 2023 Feb 14;51(5):1357–67. doi: 10.1007/s15010-023-01996-y (PMC9925936; doi:10.1007/s15010-023-01996-y)
Supplement: Supplementary file 1 — Supplementary file1 (DOCX 30 KB) [file 15010_2023_1996_MOESM1_ESM.docx]

**Supplementary Material**

**Table 1:** Rates of Underreporting

|  | **0-4 year olds** | **5-11 year olds** | **12- 17 year olds** | **Total** |
| --- | --- | --- | --- | --- |
| *JULY to OCTOBER 2021* | | | | |
| **Numbers reported to the RKI** | 957 | 517 | 695 | 2,169 |
| **Numbers reported to the DGPI** | 240 | 57 | 91 | 388 |
| **Underreporting rate** | 3.99 | 9.07 | 7.64 | 5.59 |
| *CW 09-24/2021* | | | | |
| **Numbers reported to the RKI** | 1,036 | 566 | 800 | 2,402 |
| **Numbers reported to the DGPI** | 317 | 72 | 121 | 510 |
| **Underreporting rate** | 3.27 | 7.86 | 6.61 | 4.71 |
| *CW 25-52/2021* | | | | |
| **Numbers reported to the RKI** | 2,128 | 1,497 | 1,614 | 5,239 |
| **Numbers reported to the DGPI** | 609 | 194 | 241 | 1,044 |
| **Underreporting rate** | 3.49 | 7.72 | 6.70 | 5.02 |
| *CW 01-16/2022* | | | | |
| **Numbers reported to the RKI** | 5,775 | 3,029 | 2,874 | 11,678 |
| **Numbers reported to the DGPI** | 1,968 | 610 | 423 | 3,001 |
| **Underreporting rate** | 2.93 | 4.97 | 6.79 | 3.89 |
| * Abbreviations: CW – calendar weeks; RKI – Robert Koch Institute; DGPI - German Society for Pediatric Infectious Diseases | | | | |

**Table 2:** SARS-CoV-2 seroprevalence etimates in children and adolescents (≤17y) in Germany, as determined in the SARS-CoV-2 KIDS study

N = 12243

| **Month** | **N** | **Seroprevalence** | | **95% CI** | |
| --- | --- | --- | --- | --- | --- |
|  |  | **n** | **%** | **LCL %** | **UCL %** |
| *June 2021* | 524 | 68 | 12.98 | 10.10 | 15.85 |
| *July 2021* | 406 | 57 | 14.04 | 10.66 | 17.42 |
| *August 2021* | 302 | 39 | 12.91 | 9.13 | 16.70 |
| *September 2021* | 253 | 37 | 14.62 | 10.27 | 18.98 |
| *October 2021* | 203 | 44 | 21.67 | 16.01 | 27.34 |
